# Monitoring and Prognosis System Based on the ICF for People with Traumatic Brain Injury

**DOI:** 10.3390/ijerph120809832

**Published:** 2015-08-18

**Authors:** Laia Subirats, Raquel Lopez-Blazquez, Luigi Ceccaroni, Mariona Gifre, Felip Miralles, Alejandro García-Rudolph, Jose María Tormos

**Affiliations:** 1 eHealth Department, Eurecat, Roc Boronat, 08018 Barcelona, Spain; E-Mail: felip.miralles@eurecat.org; 2 Universitat Autònoma de Barcelona, Campus UAB, 08193 Bellaterra, Spain; 3 Institut Guttmann, Institut Universitari de Neurorehabilitació adscrit a la UAB, Camí de Can Ruti, SN 08916, Badalona, Spain; E-Mails: rlopez@guttmann.com (R.L.-B.); mgifre@guttmann.com (M.G.), agarciar@guttmann.com (A.G.-R.); jmtormos@guttmann.com (J.M.T.); 4 1000001 Labs, Alzina 52, 08024 Barcelona, Spain; E-Mail: luigi@1000001labs.org

**Keywords:** international classification of functioning, disability and health, medical records, information systems, brain injuries, prognosis, public health

## Abstract

The objective of this research is to provide a standardized platform to monitor and predict indicators of people with traumatic brain injury using the International Classification of Functioning, Disability and Health, and analyze its potential benefits for people with disabilities, health centers and administrations. We developed a platform that allows automatic standardization and automatic graphical representations of indicators of the status of individuals and populations. We used data from 730 people with acquired brain injury performing periodic comprehensive evaluations in the years 2006–2013. Health professionals noted that the use of color-coded graphical representation is useful for quickly diagnose failures, limitations or restrictions in rehabilitation. The prognosis system achieves 41% of accuracy and sensitivity in the prediction of emotional functions, and 48% of accuracy and sensitivity in the prediction of executive functions. This monitoring and prognosis system has the potential to: (1) save costs and time, (2) provide more information to make decisions, (3) promote interoperability, (4) facilitate joint decision-making, and (5) improve policies of socioeconomic evaluation of the burden of disease. Professionals found the monitoring system useful because it generates a more comprehensive understanding of health oriented to the profile of the patients, instead of their diseases and injuries.

## 1. Introduction

In 1990, the World Health Organization (WHO) introduced a new measure, the disability-adjusted life years (DALYs) [1]—as a unified measure to quantify the burden of disease, injury and risk factors. The DALYs take into account the years of life lost to premature death and years of life that live in states of poor health or disability.

In this study we have worked with data from acquired brain injury (ABI) and more specifically traumatic brain injury (TBI) (I60–I69 classes of the International Classification of Diseases version 10 (ICD-10) [2]). The main cause of TBI is traffic accidents in Catalonia and 60% of those affected by TBI are under 25 years [3].

Globally, road accidents are the ninth leading cause of death with 1.3 M of deaths in 2012 [4]. The WHO projections for traffic accidents indicate that occupy seventh place in 2030 with 1.9 M of deaths (67 M DALYs). These data are particularly relevant if we consider that is expected to decrease the global burden of disease in 2030.

Under the Qvidlab [5] project, conducted by the Institut Guttmann, a committee of experts in sociology, psychology, neuropsychology, social work and neurorehabilitation of several institutions defined a set of evaluation questionnaires for social bio-psycho-profile of the people with brain injury, which form the core of periodic comprehensive evaluations (PCEs) and have the following characteristics:

- They are used to collect some information about the state of a person or an activity;
- They are validated by a health care institution;
- They are stored in the clinical record [6];
- They refer to specific problems or people;
- Their selection depends on the cultural aspects, organizations and countries.

Consequently, often the same type of information is captured by different instruments, with the consequent difficulty of interoperability. Therefore there is the need to unify the information in multiple surveys into a single classification instrument. There are minimum standardized data sets, called *core sets*, but its introduction involves changes in the way each organization represents knowledge, and these changes in the protocols established for evaluating the person can be expensive. Moreover, changes in assessment tools often result in the devaluation of the clinical records, so that organizations may be reluctant to change. For example, if instead of managing the Disability Rating Scale (DRS) we directly manage its standardization to the International Classification of Functioning, Disability and Health (ICF), the clinical records devalue because the DRS questionnaire has been designed and optimized to assess functionally people with moderate and severe traumatic brain injury. The core sets have been created as a selection of the indicators of international standards, such as ICF [7] or systematized nomenclature of Medicine—Clinical Terms (SNOMED CT) [8], relevant to people with specific diseases. There are specific studies on the basic set for ABI [9,10], on how to implement ICF [11,12,13,14] and how to represent ontologically this information [15,16,17]. On the other hand, there are several works that study the use of ICF in different domains [18], quality of life according to the context [19] and prediction and analysis of indicators in the field of TBI [20,21,22].

To encode indicators into international standards, the World Health Organization’s ICF is considered because it provides a comprehensive specification of health-related human functioning in the domains of (i) body functions and structures (e.g., sensory, neuromusculoskeletal and movement-related functions), (ii) activities and participation, ranging from basic (e.g., dressing and eating) to complex (e.g., working and living independently), and (iii) environmental factors that provide a context for understanding functioning, functional diversity and health. ICF standard has five qualitative, ordered values, plus *not specified* and *not applicable*: 0 (no), 1(mild), 2 (moderate), 3 (severe), 4 (complete), 8 (not specified), 9 (not applicable) deficiency/limitation/restriction. Considering that the ICF allows that this tool could be used for any disability, we present TBI as a use case.

Furthermore, the tool also has the potential to improve existing systems that evaluate the efficiency (cost-effectiveness) prevention programs [23]. This tool is designed to save costs and time, to enable to have more information to make decisions, to promote interoperability, to facilitate joint decision making and to improve policies for socio-economic evaluation.

Finally, regarding the clinical decision support system (CDSS) or prognosis system, there is the need to predict the next state of the indicators in order to be able to foresee possible complications. In addition, it is useful to know which attributes are relevant for the prediction and the dependence of time of indicators. The CDSS provides all this information and evaluates the prediction of the evolution of two indicators (emotional and executive functions) that are taken as examples.

## 2. Experimental Section

### 2.1. Scenario

In the rehabilitation of people who suffer from neurological diseases, there are two basic stages: *intra-hospital* and *extra-hospital*. In the intra-hospital (acute) phase, patients who suffered from a traumatic or non-traumatic injury stay in hospital undergoing rehabilitation. After typically a few months in hospital, they return home and the extra-hospital phase starts. Thereafter they go once a year to the rehabilitation hospital for a *periodic comprehensive evaluation* (PCE), when they are administered several questionnaires (depending on the disease they are suffering from) with the aim of measuring functioning independence, psychological and social variables. This study is focused on the extra-hospital or chronic phase. There is a dataset of people with ABI having as indicators demographic and clinical data. *Clinical data* varies over time and it is collected once a year.

The following example corresponds to a real prognosis case scenario of a person with ABI.

Anna is the (anonymized) name of a 43-year woman from Barcelona who suffered a head injury due to a car accident 26 years ago. After completing her regular assessment at the Institut Guttmann, a psychologist visits her and displays a graphical monitoring system that represents the state of the person individually and compared with people with the same disease. The CDSS helps her psychologist to predict problems in emotional functions and executive functions.

The questionnaires used to gather *clinical data* in the PCE for people who suffer ABI are:

- Institut Guttmann social scale (ESIG) [5], that analyzes social inclusion;
- Community integration questionnaire (CIQ) [24], that measures home integration, social integration and productive activities;
- Patient competency rating scale (PCRS) [25], that measures activities of daily living, behavioral and emotional functions, cognitive abilities, and physical functions;
- Behavioral scale, PCRSi (informer) [25], that is similar to PCRS but evaluated by an informer;
- Rancho scale levels of cognitive functioning [26], that generates a classification of the patient in one of eight levels;
- Barthel index [27], that it is an ordinal scale used to measure performance in activities of daily living (ADL);
- Disability rating scale (DRS) [28], that addresses impairment, disability and handicap; and
- Extended Glasgow outcome scale (GOSE) [29], that generates a classification of the patient in one of eight levels.

However, if necessary, other questionnaires could be added to the tool. Regarding *demographic data*, the used data in the two predictions are age, gender, years from diagnosis, disease and origin. The ABI data set contained data of 730 people, 470 male and 260 female, and from 17 to 90 years old.

### 2.2. Monitoring System

The steps that have been taken to standardize the ICF were: (1) standardization of attributes, (2) standardization of values, (3a) aggregation values and (3b) inference values from other attributes. For example, if we consider the first attribute Barthel Index, *feeding*, its standardization is *eating* (d550). Furthermore, the attribute values are 10, 5 and 0; corresponding to the standardized values no involvement (0), moderate impairment (2) and complete (4) respectively. The third attribute of the questionnaire CIQ, *competences at home/feeding*, it is also standardized to *eating* category, therefore an aggregation of values for this attribute is required. Finally, if the *self-care* category (d5) has no value, it is calculated through the average of the lower categories that have a value.

**Figure 1:**
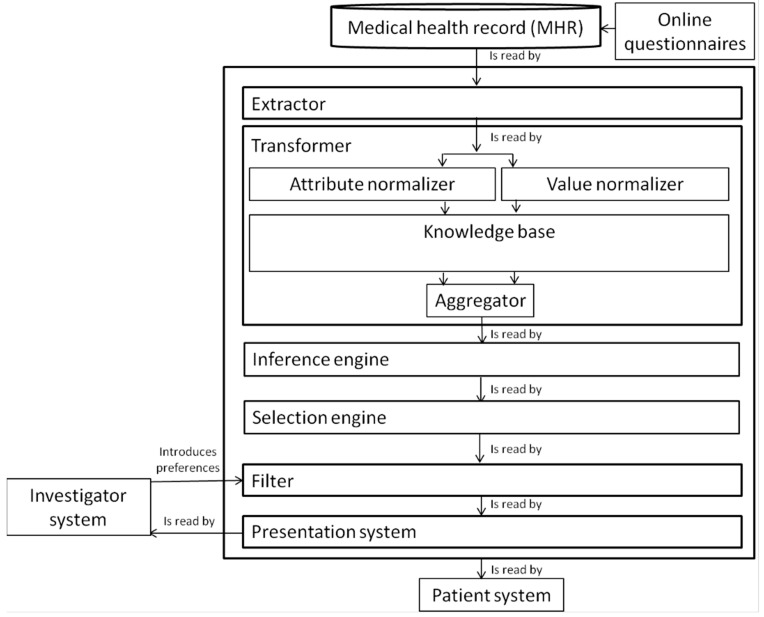
Flowchart of the automatic generation of multidimensional indicators.

We analyzed the experience of the monitoring system with five health professionals (a neuropsychologist, a doctor, a social worker and two psychologists). Quantitative and qualitative information was collected by verbal reports protocol. This monitoring system has been implemented in Liferay Portal CE, which is available under the GNU Public License (LGPL) v2.1. However, the results of this system are not available to the public and there is a published patent [30] of the standardization method. MATLAB was used for the different plots [31]. Figure 1 shows the flowchart of the standardization of the ICF and its integration in the monitoring system, which is comprised of an extractor, a transformer, an inference engine, a selector, a filter and a presentation system. The extractor access databases and selectively fetches information. The transformer performs the standardization of attributes and values (performed by the attribute normalizer and value normalizer respectively) and aggregates the information. Then, the inference engine performs when necessary the inference of values from other attributes. The selector selects the relevant information depending on the particular profile being analyzed. The filter enables the personalization of data visualization enabling filtering by a plurality of parameters. Finally, the presentation system enables the graphical representation of all normalized data.

### 2.3. Clinical Decision Support System (CDSS)

Only patients with at least 3 years of measurements and who had a not empty value in the last measurement were taken into account in the CDSS (see in Table 1 that the minimum length of the time series is 3 and that the percentage of missing values of predicted values in 2013 is 0%). This is due to the fact that the last measurement is used to validate the prediction. As a consequence, used data in the CDSS is summarized in Table 1.

Regarding the prognosis system, existing temporal representation and reasoning methods are compared using Weka. Weka is used to compute the different reasoning algorithms because it is an open-source tool that provides a user-friendly GUI that allows evaluating the predictions and obtaining output predictions easily. Two representation methods are used: the full time-series and the previous state of the time-series. On the other hand, the chosen algorithms to perform the benchmark are: k-nearest neighbor (KNN), Naïve Bayes (NB), support vector machine (SVM) and J48. Given that in a confusion matrix we have true positives (TP), false positives (FP), true negatives (TN) and false negatives (FN) we define:

$$accuracy=\frac{TP+TN}{TP+FP+FN+TN},precision=\frac{TP}{TP+FP},recall=\frac{TP}{TP+FN},specificity=\frac{TN}{TN+FP}$$

In order to see the most important indicators for the prediction, BigML [32] (a machine learning service) was used as it provides a comprehensive report of relevant attributes for the prediction.

**Table 1:** Demographic and clinical data of the prognosis of disabilities of neurological origin with acquired brain injury (ABI) in the clinical decision support system (CDSS).

AttributePrognosis	Emotional Functions (419 people)	Executive Functions (477 people)
Age	(17,90), mean = 46.7 stdDev = 15.5	(17,90), mean = 47.2 stdDev = 15.6
Gender	female (145), male (274)	female (164), male (313)
Years from diagnosis	(4,67), mean = 17.9 stdDev = 15.7	(2,72), mean = 19.0 stdDev = 17.0
Disease	Not assigned (2), Guillain-Barre (18), polio (14), plexus (5), mielomeningocele (20), traumatic brain injury (213), multiple sclerosis (43), other progressive diseases (22), children cerebral palsy (103), hemorrhagic stroke (122), thrombotic stroke (26), embolic stroke (12), undetermined ischemic brain stroke (24), other ischemic brain stroke (9), other degenerative diseases not traumatic (84), muscular dystrophy (1), poliradiculoneuritis (7), other (4)	Not assigned (2), Guillain-Barre (14), polio (7), plexus (3), mielomeningocele (14), traumatic brain injury (133), multiple sclerosis (23), other progressive diseases (13), children cerebral palsy (73), hemorrhagic stroke (87), thrombotic stroke (22), embolic stroke (10), undetermined brain stroke (14), other ischemic brain stroke (5), other degenerative diseases not traumatic (51), muscular dystrophy (1), poliradiculoneuritis (3), other (2)
Origin	Traumatic (131), medic (208), undefined (80)	Traumatic (134), medic (231), undefined (112)
Length of the time series	(3,7), mean = 4.2, stdDev =1.0	(3,7), mean = 4.2, stdDev = 1.0
Missing values of the time series in the predicted attribute	2007 (99%), 2008 (80%), 2009 (54%), 2010 (42%), 2011 (39%), 2012 (52%), 2013 (0%)	2007 (77%), 2008 (69%), 2009 (69%), 2010 (45%), 2011 (37%), 2012 (47%), 2013 (0%)
Prediction	No deficiency (120), mild deficiency (130), moderate deficiency (112), severe deficiency (39), complete deficiency (18)	No deficiency (100), mild deficiency (69), moderate deficiency (103), severe deficiency (91), complete deficiency (114)

## 3. Results

The **monitoring system** calculates the evolution of different indicators and allows monitoring data representing each person individually or in the context of the group to which it belongs considering his/her disease.

### 3.1. Individual Representation

Consider the 43-year-Barcelona woman who suffered a head injury due to a car accident 26 years ago. After completing her regular assessment at the Institute Guttmann, psychologist visits her and displays a graphical monitoring system representing the state of the person individually and compared with people with the same disease.

**Figure 2:**
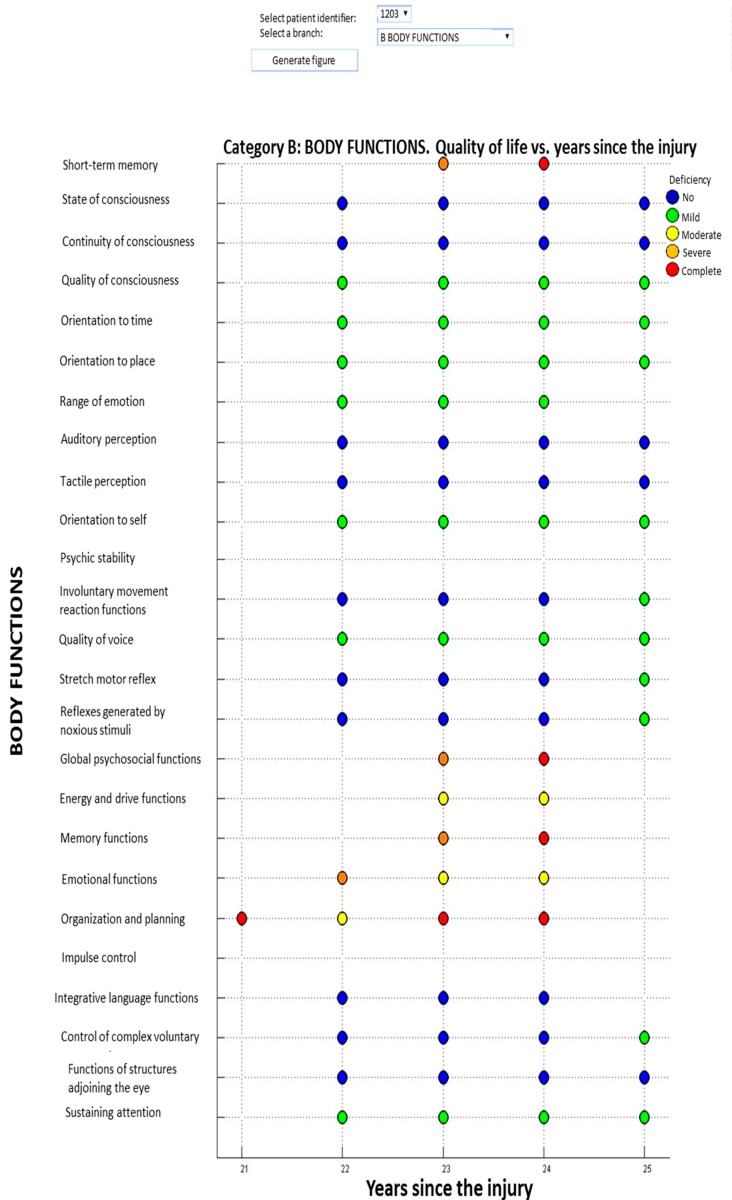
Graphical representation of the evolution of an individual through International Classification of Functioning, Disability and Health (ICF) categories.

Figure 2 represents information related to the evolution of the health of the person, represented as a set of indicators based on the elapsed time from injury. Values of limitation, deficiency or restriction are represented as follows: red/4 complete level, orange/3 severe level, yellow/2 moderate level, green/1 mild level, and blue/0 no level for of any limitation, deficiency or restriction. The professional can quickly distinguish in red those categories with greater impairment.

### 3.2. Population Representation (TBI Population)

**Figure 3:**
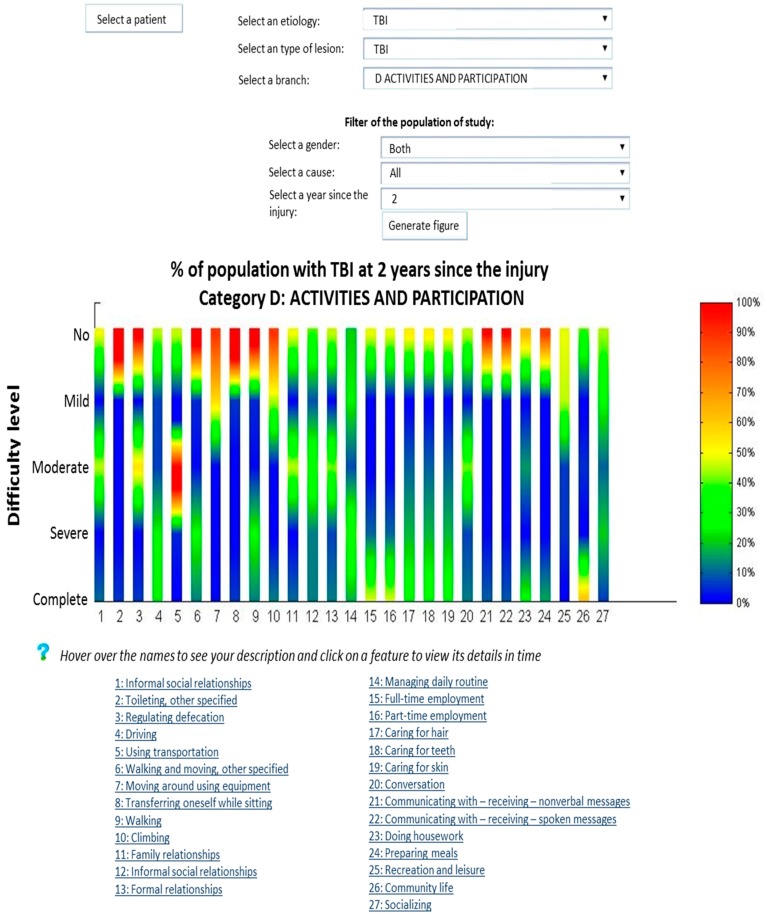
Graphical representation of the situation of the population of traumatic brain injury (TBI) and individuals through the ICF categories.

**Figure 4:**
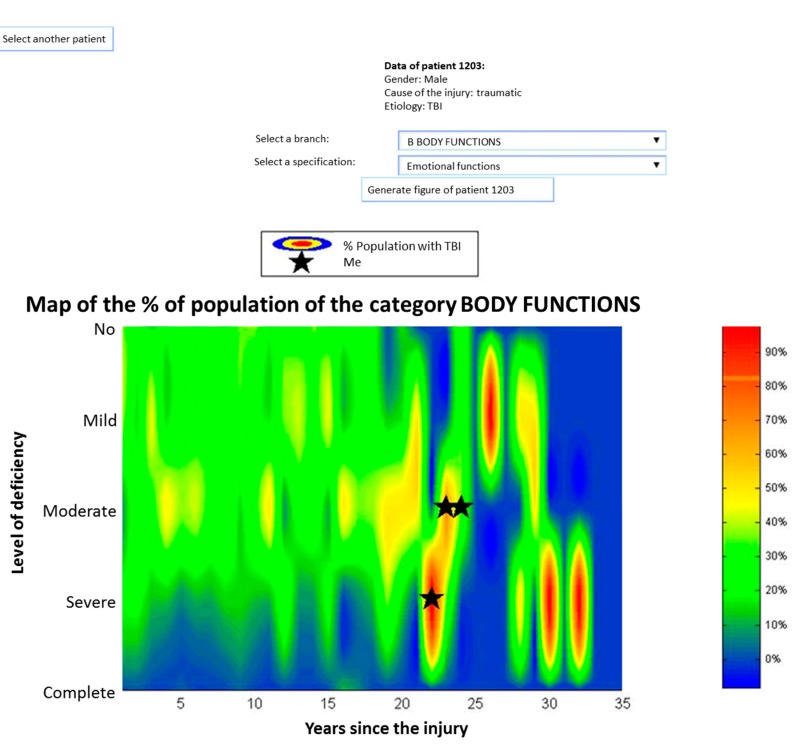
Graphical representation of the evolution of a population with TBI through ICF categories.

Figure 3 shows a representation of 32 people with TBI. These graphs show, with different colors, the percentage of the population with a certain value of deficiency of different indicators, in an instant of time. Regarding the evolution of the emotional functions of the person, in Figure 4 an improvement from moderate to severe deficiency is appreciated. This representation allows a comparison between him and the TBI population of 30 people suffering similar problems.

Regarding the CDSS, in the following tables (Table 2), the method with the best value of accuracy is highlighted with a grey background, and best values of accuracy, precision, recall and specificity are highlighted in bold.

**Table 2:** Prediction of emotional functions of people with ABI.

Temporal Representation	Learning	Accuracy	Precision	Recall (or Sensitivity)	Specificity
Full time-series	KNN (k = 7)	0.33	0.35	0.33	0.82
Full time-series	NB	0.37	0.38	0.37	0.79
Full time-series	SVM	0.41	0.41	0.41	0.85
Full time-series	J48	0.38	0.34	0.37	0.84
Previous state	J48	0.37	0.32	0.35	0.77

Using BigML, we obtain the ten most relevant attributes for the classification ordered by importance (the used notation is the ICF category followed by its code in brackets, followed by the year of the measurement and followed by the % of importance):

(1). Organization and planning (b1641) 2012: 51.93%
(2). Dressing (d540) 2012: 12.29%
(3). Personal economic resources (d8700) 2012: 6.75%
(4). Eating (d550) 2012: 5.57%
(5). Age: 3.56%
(6). Toileting other specified (d5308) 2011: 3.19%
(7). Carrying out daily routine (d230) 2010: 2.78%
(8). Family relationships (d760) 2011: 2.58%
(9). Recreation and leisure (d920) 2012: 2.21%
(10). Sports (d9201) 2009: 1.77%

Regarding the prediction of executive functions, Table 3 shows an example of a data set where the previously stated approach obtains the best values of accuracy, precision, recall and specificity.

**Table 3:** Prediction of executive functions of people with ABI.

Temporal Representation	Learning	Accuracy	Precision	Recall (Or Sensitivity)	Specificity
Full time-series	KNN (k = 7)	0,32	0,30	0,32	0,82
Full time-series	NB	0,43	0,42	0,43	0,86
Full time-series	SVM	0,40	0,41	0,40	0,85
Full time-series	J48	0,42	0,34	0,42	0,84
Previous state	J48	0,48	0,47	0,48	0,87

Using BigML, we obtain the ten most relevant attributes for the classification ordered by importance:

(1). Organization and planning (b1641) 2011: 56.40%
(2). Personal care providers and personal assistants (e340) 2011: 20.26%
(3). Work and employment other specified and unspecified (d859) 2011: 8.08%
(4). Acquiring a place to live (d610) 2012: 7.61%
(5). Complex economic transactions (d865) 2011: 1.96%
(6). Informal education (d810) 2011: 1.32%
(7). Organization and planning (b1641) 2012: 1.21%
(8). Apprenticeship (work preparation) (d840) 2011: 1.06%
(9). Full-time employment (d8502) 2011: 0.79%
(10). Sports (d9201) 2009: 1.77%

We can see that organization and planning plays an important role in the prediction of both emotional and executive functions.

## 4. Discussion

All professionals noted that the plots of the evolution of individuals and populations provide valuable visual information for understanding health status. 80% of users with disabilities gave special importance to the ability to access information at any time. Although this version is aimed at Spanish-speaking countries, it can be easily extended internationally. This would be useful for governments, and which would provide an overview of disability and a comparison between different regions. Also, it would allow the creation of best practices and common recommendations at European, national and regional level with a direct impact on regional policy of inclusion, support and assistance to dependence. In addition, the potential value of using this system is monitoring and predicting the PCEs through use cases exposed. The main beneficiaries’ actors are summarized in Table 4.

**Table 4:** Analysis of the potential value of the monitoring and prognosis system

ValueMain Target	Person with Disabilities	Professional	Health Center	Administration
Saving time	X	X		
Saving costs			X	
More information to make decisions		X		
Interoperability	X	X	X	X
Joint decision making	X	X		
Improving the socio-economic evaluation				X

### 4.1. Saving Costs and Time

By digitalizing the monitoring of individuals, professionals can administer questionnaires and follow up from computers, tablets and phones.

Use Case: A disabled person moves to Guttmann Institute for the PCE. While in the waiting room, responds to the self-administered questionnaire with a tablet provided by the professional.

### 4.2. Having More Information to Make Decisions

New knowledge is generated through reports and individual/population and static/evolution plots. Achieving therapeutic goals is assessed from a holistic and standard description.

Use Case: A person performs a process of improving activities of daily living. Using the new generation of knowledge, professionals see that has not reached therapeutic goals in a number of categories ICF values. This representation allows you having more knowledge on improving the individual and his/her level of functionality.

### 4.3. Promoting Interoperability

With the use of ICF, interoperability with other institutions using different questionnaires or languages are easier.

Use Case: After suffering a car accident, a family of Russia decides to go to Spain for rehabilitation. After making the rehabilitation process, the family wants to show its progress in its monitoring center in Moscow. Using the proposed system, the monitoring is standard and easy.

### 4.4. Facilitating Joint Decision Making

The proposed system can help clinicians make decisions together with people with disabilities. In some cases professionals can use the population representations to convince people assisted to make decisions.

Use case: a neuropsychologist sport often recommended, but the person does not see the point. The doctor uses a graph to show how indicators improve quality of life in people with a similar profile that follow their recommendation. The person is convinced that sport should be performed.

### 4.5. Improving Policies for Socio-Economic Assessment of Disease Burden

Through a specific module, the proposed system allows to calculate the evolution of these socio-economic throughout the process of rehabilitation [33] indicators:

- quality-adjusted life years (QALYs) - that are earned, calculated from the ICF indicators;
- DALYs averted, calculated from the QALYs; and
- savings calculated from the cost of DALYs averted minus the cost of the rehabilitation process.

Use Case: The Agency for Health Quality and Evaluation of Catalonia (*AQuAS*) wants to analyze the socioeconomic impact of rehabilitation. Through economic evaluation module monitoring system, they can see the QALYs gained on average in each rehabilitation process. They can also see the average of DALYs averted and the cost of each rehabilitation process. Finally, the evaluation of public policies *AQuAS* can see the cost savings assumed by being in rehab.

The system has several limitations such as the amount of data. When looking at the evaluation of the prognosis system, having more data would improve its performance. To increase the amount of data, other institutions and countries could integrate their data to the monitoring and prognosis system. Therefore, if these institutions use other questionnaires, they should add them to the tool together with their translation to the ICF.

In conclusion, this study shows the potential benefits of using ICF standard in monitoring people with TBI. It also highlights the need for a larger set of data, and integration of knowledge from different institutions and countries.

## 5. Conclusions

There are methodologies in the field of neurological disability and other areas, to automatically standardize indicators of health status according to the International Classification of Functioning, Disability and Health. This monitoring and prognosis tool allows comparing different indicators of health care facilities. The study introduces and analyzes a monitoring and prognosis tool for people with TBI, and the new knowledge generated in the form of standard indicators. This tool saves costs and time, enables to have more information to make decisions, promotes interoperability, facilitates joint decision making and improves policies for socio-economic evaluation.
